# Supplementary material for: Aptardi predicts polyadenylation sites in sample-specific transcriptomes using high-throughput RNA sequencing and DNA sequence
Source: Nat Commun. 2021 Mar 12;12:1652. doi: 10.1038/s41467-021-21894-x (PMC7955126; doi:10.1038/s41467-021-21894-x)
Supplement: Supplementary file 1 — Supplementary Information [file 41467_2021_21894_MOESM1_ESM.pdf]

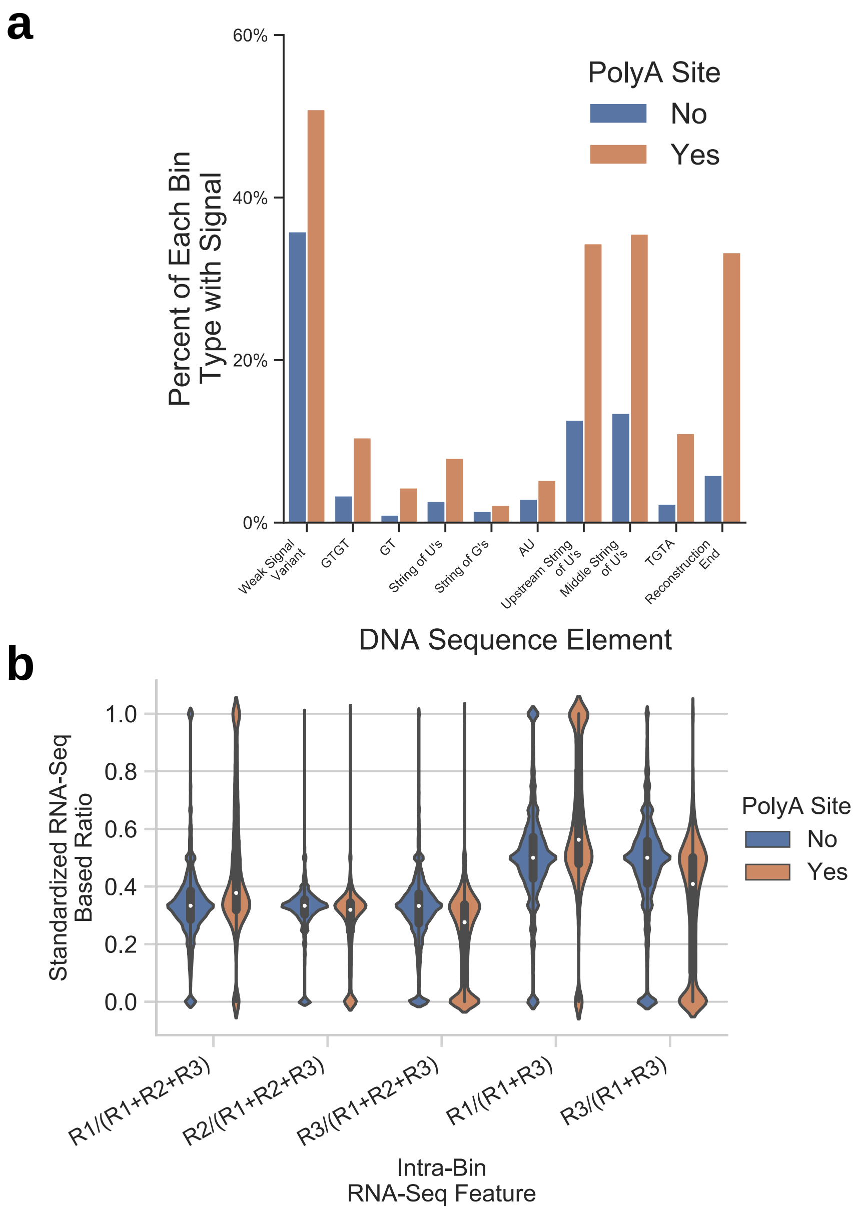

Supplementary Fig. 1: DNA sequence and RNA sequencing (RNA-Seq) features for each bin as a function of whether the bin contains a polyadenylation (polyA) site. a, The percent of 100 base bins containing the listed DNA sequence feature stratified by the bin not containing (blue) or containing (orange) a polyA site. **b,** Distribution of the standardized ratios for the intra-bin RNA-Seq features for each 100 base bin stratified by the bin not containing (blue) or containing (orange) a polyA site (each RNA-Seq ratio feature was standardized using the training set). Data shown are from the Human Brain Reference dataset.

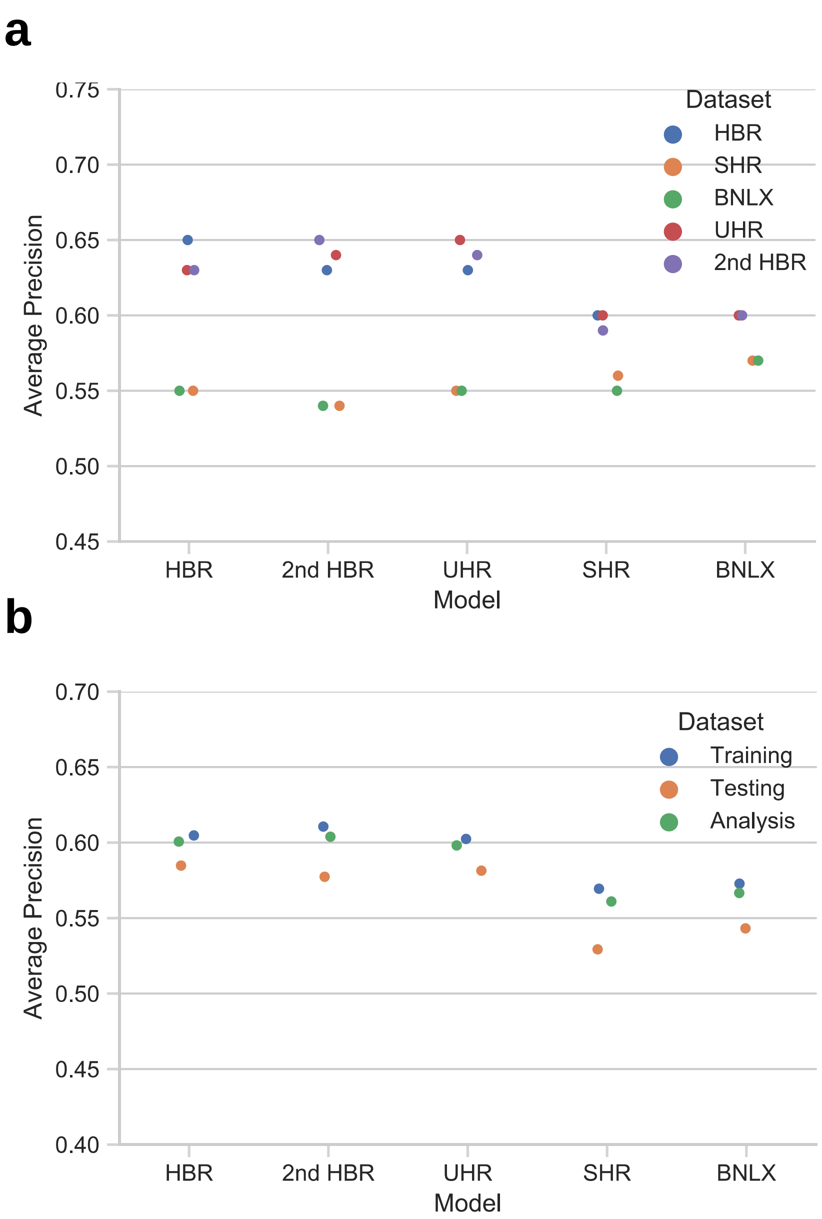

Supplementary Fig. 2: The machine learning pipeline used to build aptardi is robust to different datasets. a, Prediction models built on a given dataset perform comparably across all datasets. Colors denote the dataset used to build the predictive model, and the x-axis indicates the model used to calculate the average precision (y-axis) on the given dataset. **b**, Model performance is consistent similar the training, testing, and analysis (entire dataset without merging modified 3' terminal exons) sets.

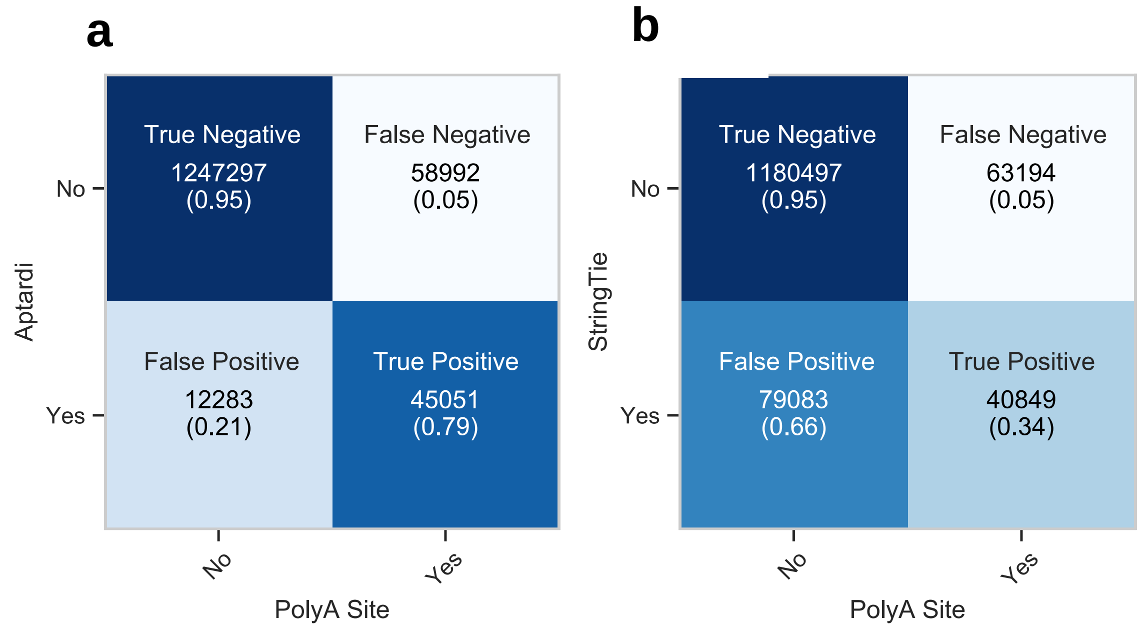

Supplementary Fig. 3: Aptardi improves the classification confusion matrix compared to StringTie. a, The confusion matrix from the aptardi prediction model generated from the Human Brain Reference (HBR) dataset improved the positive predictive value by increasing the proportion of true positive tests among positive aptardi results compared to **b,** the confusion matrix from StringTie on the same dataset. Classifications on each 100 base increment (i.e. bin) included in the analysis were compared. For the aptardi prediction model, its predictions for the presence (Yes) or absence (No) of a polyadenylation (polyA site) site were determined using the default probability threshold (0.5). For StringTie, the presence or absence of any 3' terminus within the bin from its transcriptome was used as positive and negative predictions, respectively. True polyA sites were taken from the HBR PolyA-Seq data.

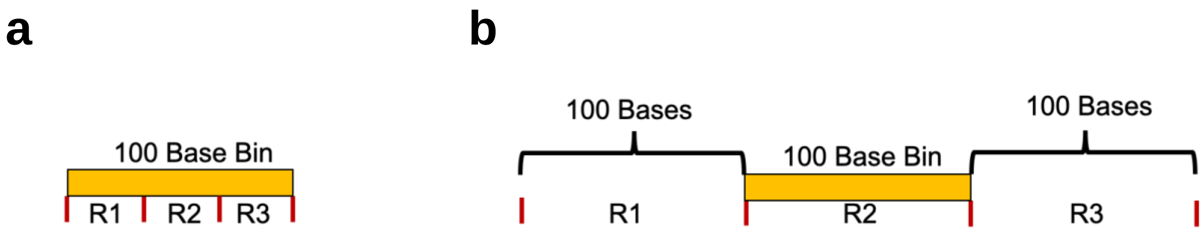

Supplementary Fig. 4: Simple depiction of the a, intra- and b, inter-bin comparisons used to engineer RNA sequencing features. For the **a,** intra-bin comparison, the bin of interest (default 100 bases) was divided into three roughly equally sized regions – R1, R2, and R3 – representing the beginning, middle, and end region of the bin, respectively. For the **b,** inter-bin comparisons, the bin of interest was considered R2, and the 100 bases upstream and downstream the bin were considered R1 and R3, respectively.

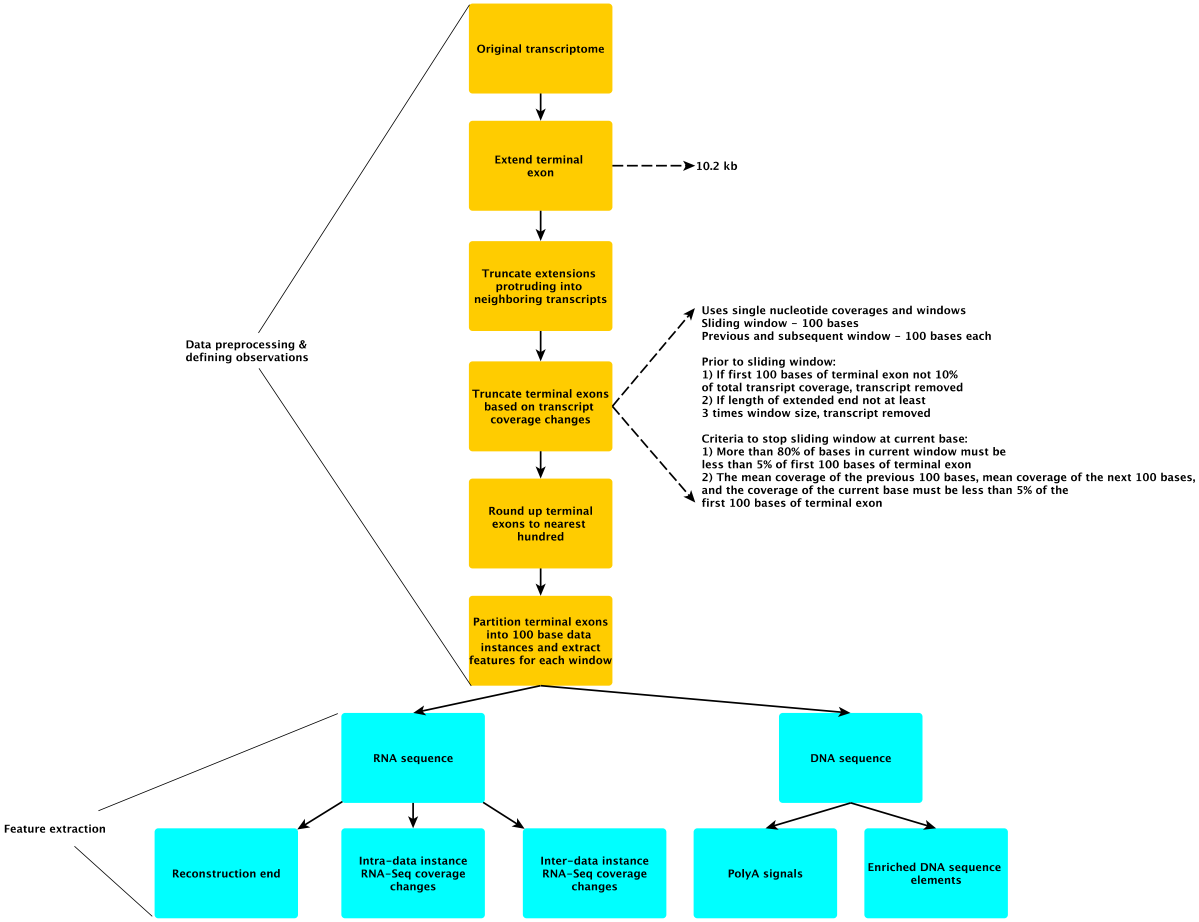

Supplementary Fig. 5: The data processing pipeline used by aptardi prior to machine learning.

The 3’ terminal exons of input transcripts are processed by aptardi (yellow) followed by feature extraction (blue).

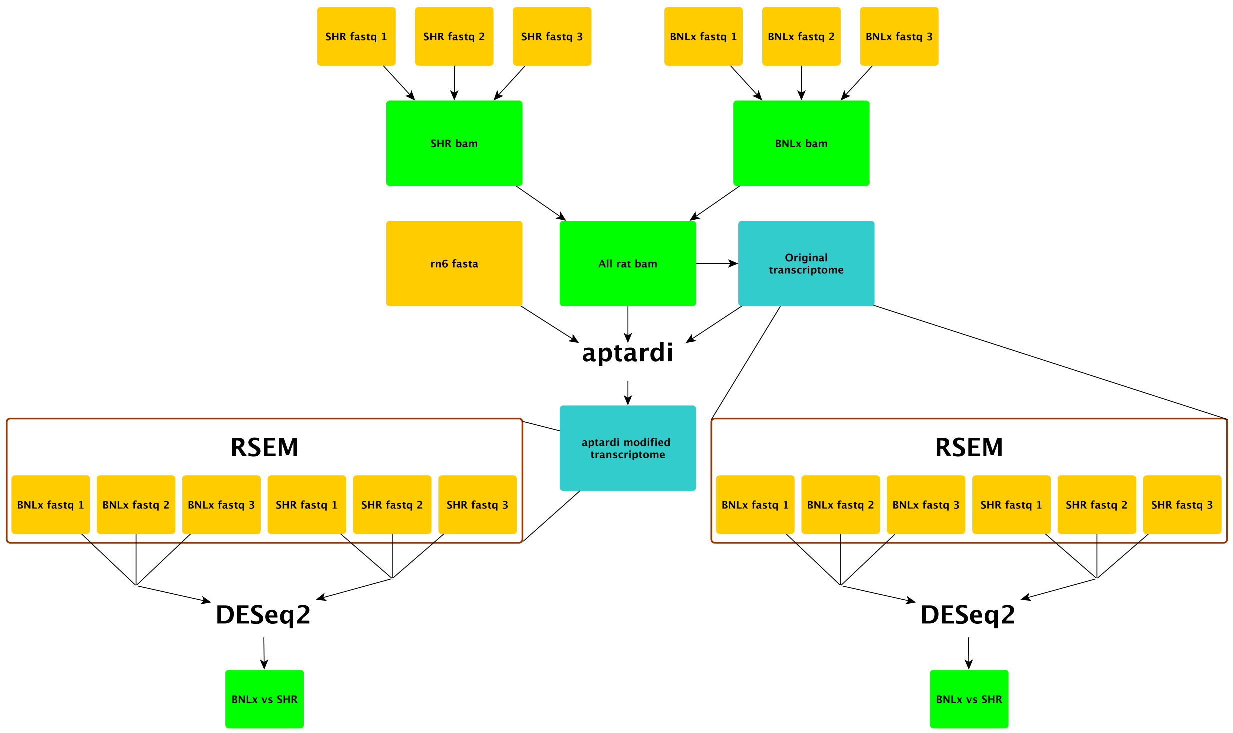

Supplementary Fig. 6: Flowchart depicting the differential expression analysis between the two inbred rat strains, BNLx and SHR. Yellow boxes denote raw data, green boxes denote data that we generated, and blue boxes denote the two transcriptomes separately subjected to RSEM (RNA-Seq by Expectation Maximization) for comparison.

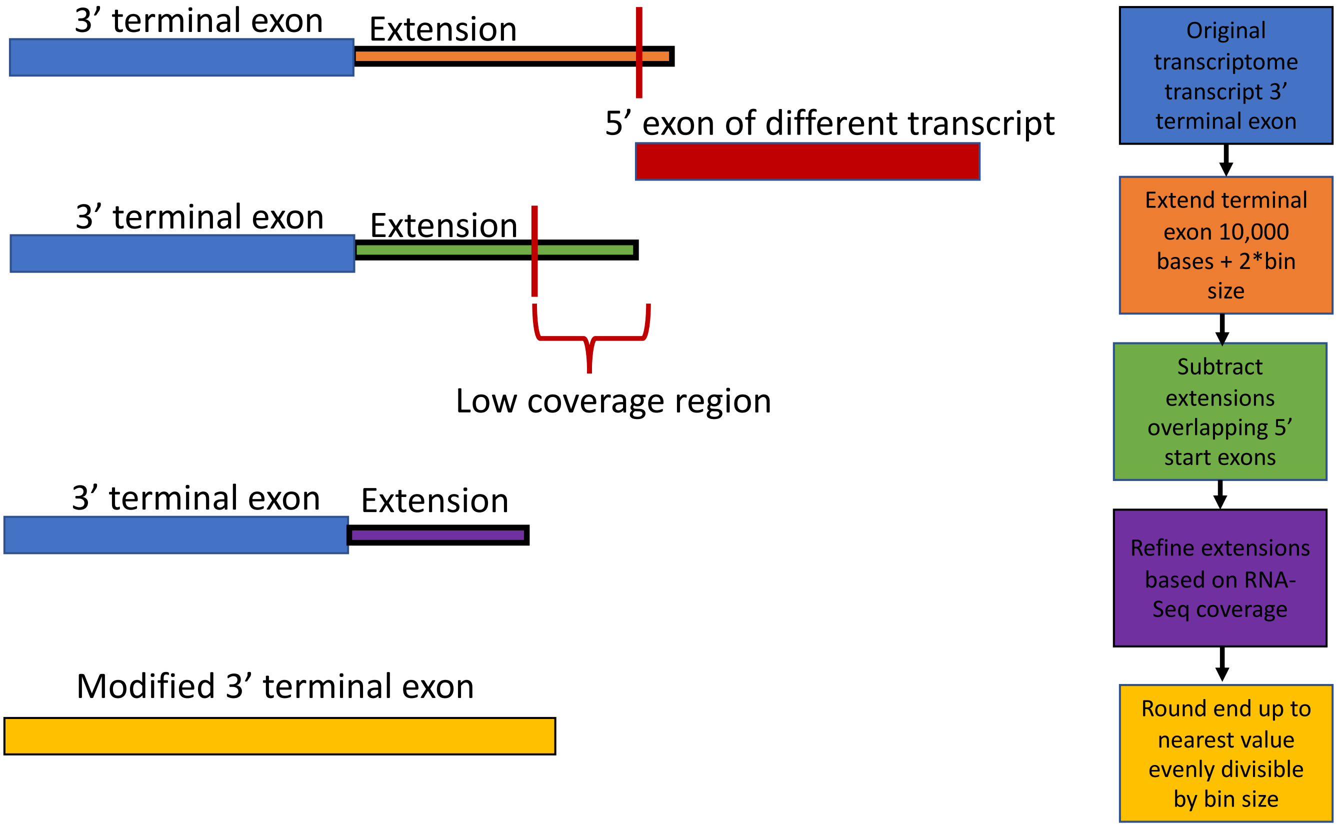

Supplementary Fig. 7: Graphical depiction of the transcript processing steps. The 3’ terminal exon of a transcript derived from the original transcriptome (blue) is first extended 10,000 bases plus two times the bin size (orange). If the extension overlapped the 5’ exon of a neighboring transcript on the same strand (red), the extension was reduced to remove the overlap (green). Next the RNA-sequencing (RNA-Seq) coverage at single nucleotide resolution was used to shorten the 3’ terminal exon to only include regions with detectable coverage relative to the start of the 3’ terminal exon (purple). Finally, the 3’ terminal exon was rounded up to the nearest value evenly divisible by the bin size for compatibility with machine learning (yellow).

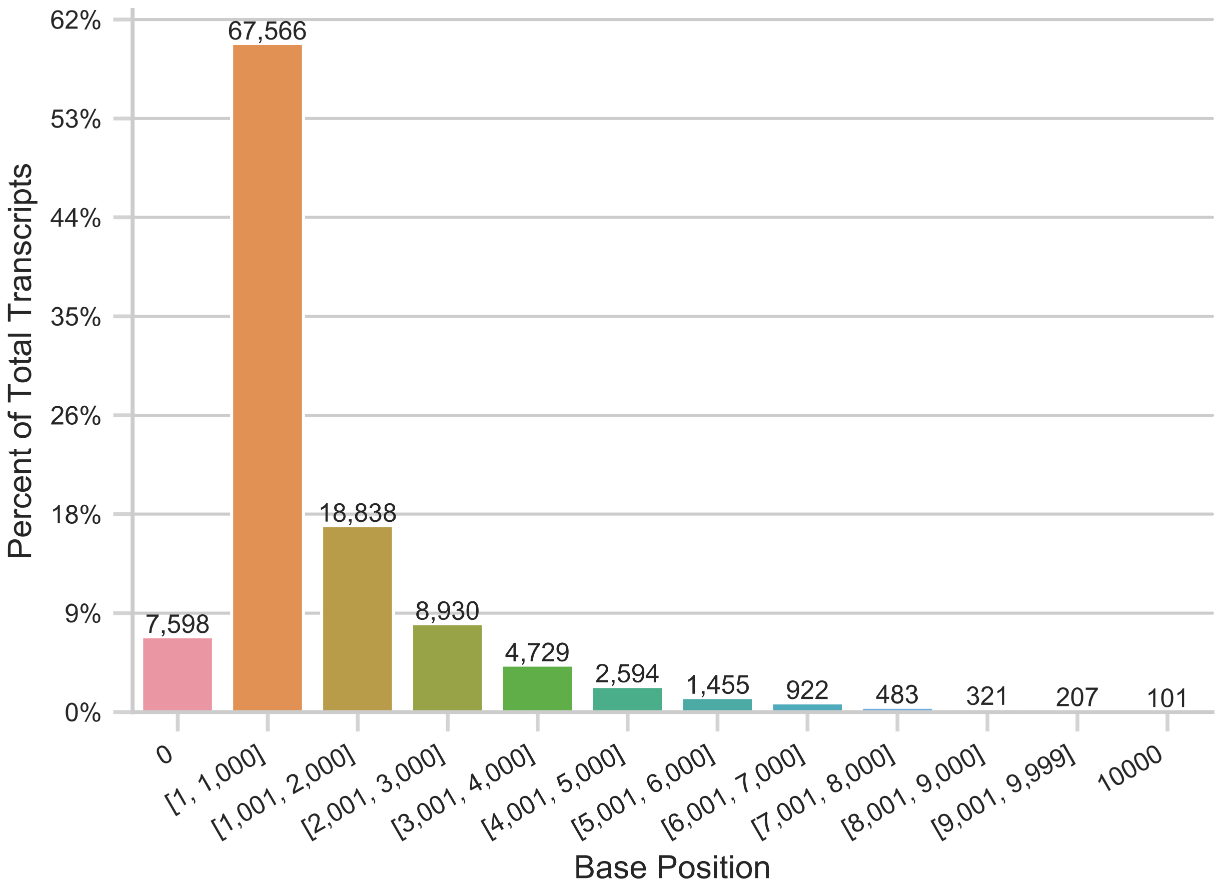

Supplementary Fig. 8: Results from truncating modified 3' terminal exon extensions based on transcript coverage. Transcripts were shortened based on coverage as described in Transcript processing section of Methods. The base position relative to the start of the terminal exon is given on the x-axis. Over half the modified 3' terminal exons were shortened to <= 1,000 bases. A base position value of zero indicates the transcript was removed entirely because its modified 3' terminal exon did not meet the minimum coverage requirements. Data shown are from the Human Brain Reference dataset.

Supplementary Table 1: Datasets used to evaluate aptardi.

	RNA				DNA Source	True Polyadenyation Sites Source
Dataset	Source	Read Length	Stranded?	# Reads		
HBR	Human Brain Reference	100	Yes	115,926,448	hg38/GRCh38	PolyA-Seq Human Brain Reference Total RNA
2nd HBR	Human Brain Reference	75	Yes	139,851,362	hg38/GRCh38	PolyA-Seq Human Brain Reference Total RNA
UHR	Universal Human Reference	75	Yes	145,513,666	hg38/GRCh38	PolyA-Seq Universal Human Reference Total RNA
SHR	SHR Inbred Rat Brain	100	No	111,812,107	SHR Strain Specific	PolyA-Seq Sprague Dawley Total RNA
BNLx	BNLx Inbred Rat Brain	100	No	74,863,513	BNLx Strain Specific	PolyA-Seq Sprague Dawley Total RNA

Supplementary Table 2: Comparison of the positive predictive value (PPV) and number of polyadenylation (polyA) sites annotated between the original transcriptome, aptardi modified transcriptome, TAPAS[1], and APARENT[2] at different base distance cutoffs and utilizing different polyA site annotation databases. A prediction was considered a true positive if it was within the given base distance cutoff of an annotated polyA site. Annotated polyA sites were taken from the human brain reference (HBR) PolyA-Seq data, PolyASite 2.0[3], and PolyA_DB[4]. The original transcriptome was generated from the HBR dataset, and predictions by aptardi, TAPAS, and APARENT were made using these transcript structures. Namely, TAPAS used the HBR RNA-Seq data, APARENT used the hg38/GRCh38 reference human genome, and aptardi used both.

	Source of PolyA Sites	Base Distance Cutoff	True Positives	False Positives	PPV	Number of PolyA Sites Annotated
Original Transcriptome	HBR PolyA-Seq	100	39,842	74,081	0.35	23,685
Aptardi Modified Transcriptome			62,688	79,088	0.44	29,327
TAPAS			22,804	51,810	0.31	25,180
APARENT			33,213	238,883	0.14	27,999
Original Transcriptome		50	35,731	78,192	0.31	19,511
Aptardi Modified Transcriptome			49,025	92,751	0.35	23,192
TAPAS			18,357	56,257	0.25	19,064
APARENT			23,562	248,534	0.09	22,153
Original Transcriptome		25	30,761	78,192	0.28	16,236
Aptardi Modified Transcriptome			38,044	103,732	0.27	18,226
TAPAS			14,303	60,311	0.19	14,281
APARENT			19,560	252,536	0.08	18,371
Original Transcriptome	PolyASite 2.0	100	51,191	62,712	0.45	45,562
Aptardi Modified Transcriptome			76,277	65,452	0.54	54,925
TAPAS			33,481	41,133	0.45	51,286
APARENT			73,232	198,864	0.37	80,512
Original Transcriptome		50	44,249	69,654	0.39	31,861
Aptardi Modified Transcriptome			60,900	80,829	0.43	37,842
TAPAS			26,418	48,196	0.35	33,567
APARENT			54,665	217,431	0.25	59,115
Original Transcriptome		25	36,996	76,907	0.32	21,969
Aptardi Modified Transcriptome			46,973	94,756	0.33	25,218
TAPAS			20,063	54,551	0.27	20,743
APARENT			43,722	228,374	0.19	42,425
Original Transcriptome	PolyA_DB	100	49,648	64,255	0.44	41,893
Aptardi Modified Transcriptome			75,531	66,198	0.53	51,381
TAPAS			31,379	43,235	0.42	46,125
APARENT			63,249	208,847	0.30	66,770
Original Transcriptome		50	43,960	69,943	0.39	30,717
Aptardi Modified Transcriptome			61,348	80,381	0.43	36,974
TAPAS			25,319	49,295	0.34	31,369
APARENT			47,852	224,244	0.21	50,794
Original Transcriptome		25	37,670	76,233	0.33	22,340
Aptardi Modified Transcriptome			48,112	93,617	0.34	25,833
TAPAS			19,482	55,132	0.26	20,307
APARENT			40,149	231,947	0.17	38,965

Supplementary Table 3: RNA sequencing alignment results for mouse tissue analysis. Reads were aligned to the mm10/GRCm38 mouse reference genome with HISAT2 (v.2.1.0).

Dataset	Brain	Liver
# Reads	15,239,319	15,991,252
Overall Genome Alignment Rate	98.70%	97.46%

Supplementary Table 4: Few polyadenylation (polyA) sites share a 100 base region with another polyA site. Since aptardi makes predictions in 100 base increments, sites within 100 bases of one another cannot be distinguished. Data shown are from the Human Brain Reference dataset.

Total # PolyA Sites Captured	# PolyA Sites Sharing 100 Base Bin	# Multi PolyA 100 Base Bins
42,977	3,625	1,807

Supplementary Table 5: RNA sequencing alignment results for each sample. Reads were aligned to each sample's respective genome with HISAT2 (v. 2.1.0).

Dataset	HBR	2nd HBR	UHR	BNLx	SHR
# Reads	115,926,448	139,851,362	145,513,666	74,863,513	111,812,107
Overall Genome Alignment Rate	96.58%	95.39%	95.38%	96.41%	96.76%

Supplementary Table 6: RNA sequencing alignment results for the CFIm25 knockdown analysis. Reads were aligned to the hg38/GRCh38 human reference genome with HISAT2 (v. 2.1.0).

Dataset	Control	CFIm25 Knockdown
# Reads	164,774,179	160,083,915
Overall Genome Alignment Rate	94.94%	95.91%

Supplementary Table 7: The transcript processing steps increase the number of polyadenylation sites included in aptardi analysis. The number of unique polyadenylation sites captured at each step is shown, along with the category from which the site was derived.

Transcript Processing Step	# Polyadenylation Sites from Source		
	Transcript Terminal Exon	Transcript Extension	Both a Transcript's Terminal Exon and a Separate Transcript's Extension
Terminal Exon + Extension (Original)	24,640	24,107	20,707
Subtract Overlapping Starts	24,635	22,356	10,336
Truncate Based on Coverage	20,072	6,189	8,097
Window (Final)	20,541	7,437	8,390

Supplementary Table 8: Summary of engineered DNA sequence features.

DNA Sequence Element	Nucleotide String(s)	Window Size	Region Probed, if PAS Present (Relative to PAS)	Region Probed, if PAS not Present (Relative to Bin Start (for Start), Bin End (for End))	Frequency of String Required for Enrichment (>=)
Distal downstream G-rich region	>=5 G's	6	+43 to +143 (or end*)	+30 to end*	0.0585
Proximal downstream T-rich region	TTT	3	+13 to +76	+10 to +40	0.125
Proximal downstream GT/TG-rich region	GT & TG	2			0.25
Proximal downstream GTGT/TGTG-rich region	GTGT & TGTG	4			0.0469
Intermediate T-rich region	T	1	+6 to +36	-36 to 0	0.375
Upstream T-rich region	T	1	-50 to 0	-86 to -7	0.375
Upstream TGTA/TATA-rich region	TGTA & TATA	4	-40 to 0	-76 to -7	0.0469
AT-rich region	AT	2	-93 (or start*) to +142 (or end*)	Start* to end*	0.125

*Start = 100 bases upstream bin start, end = 100 bases downstream bin end

Supplementary Methods

Transcript processing.

Modified 3’ terminal exons were refined using an approach similar to that described by Ye et al.[5] and Miura et al.[6] as follows. If the average coverage of the first X bases (X = bin size) of the modified 3’ terminal exon was less than 10% of the entire transcript’s average coverage and/or the modified 3’ terminal exon was not at least three times the bin size (default 100 bases), the transcript was removed. Otherwise the transcript’s modified 3’ terminal exon was scanned 5’ to 3’ using a sliding window equal to the bin size until the following metrics were less than 5% of the average coverage of the first bases equal to the bin size of the modified 3’ terminal exon: 1) 80% of the bases in the current bin, 2) the average coverage of the previous bin, 3) the average coverage of the subsequent bin, and 4) the coverage of the current base (i.e. first base in the current bin). This strategy is robust to poor local coverage that can occur in RNA-Seq data (e.g. GC bias). The base that meets these criteria defines the end of the modified 3’ terminal exon for the transcript, i.e. this base is not considered a transcript stop site but rather defines the 3’ end of the region that will be explored by aptardi. For compatibility with machine learning, where predictions are made on a set bin size (i.e. 100 base bins as the default), each modified 3’ terminal exon was rounded up to the nearest value evenly divisible by the bin size at the 3’ end. Supplementary Fig. 7 graphically depicts these transcript processing steps. Note that since the coverage of the current and subsequent bins are used when refining modified 3’ terminal exons, the longest possible 3’ modified terminal exon is two times the bin size less than its total length.

To evaluate the impact of transcript processing on the original transcriptome fed to aptardi, we first ascertained the number of unique polyadenylation (polyA) sites captured at each step and further determined from which of the following three categories each was derived: 1) the original reconstruction terminal exon, 2) the extension step, or 3) both (1) and (2) as a result of overlaps (Supplementary Table 8). The extension step doubled the number of polyA sites captured. After subtracting extensions overlapping a neighboring transcript's start, the number of polyA sites in (3) was halved. This suggests the extension step resulted in extensions long enough to encompass entire neighboring transcripts, supporting the need to subtract overlap. Shrinking extension length once again based on transcript coverage (see Transcript processing section in Methods) reduced the number of polyA sites captured in (2) by more than a third and removed 7,598 transcripts from analysis (Supplementary Fig. 8). This decrease is large but likely necessary to ensure polyA sites captured by a given transcript plus extension confidently belong to that extension and is being expressed. Overall, more than 7,000 novel transcript stop sites were included in aptardi analysis though transcript processing.

DNA sequence features.

All DNA sequence features were encoded as binary indicators to indicate presence (1) or absence (-1) in each bin (default 100 bases).

For each of the four polyadenylation signals (PAS's) – 1) AATAAA, 2) ATTAAA, 3) AGTAAA and any of 4) AAGAAA, AAAAAG, AATACA, TATAAA, GATAAA, AATATA, CATAAA, AATAGA – a sliding six base window was scanned from -35 bases upsteam the bin start to -7 bases upstream the

base end in single nucleotide increments. If any single hexamer matched the given PAS, it was encoded 1, otherwise -1.

In general, the 100 bases upstream and downstream the bin, as well as the bin itself (300 bases total for the default 100 base bin size) were used for the DNA sequence elements features; however, the specific region examined for each DNA sequence element varied by the given feature and whether a PAS was present. If more than one PAS was present, the PAS that dictated the region probed was first by priority in the order listed above, i.e. if AATAAA and ATTAAA were present, the location of AATAAA was used, and next by the first occurrence of the location, i.e. if AATAAA was present multiple times, the location of the 5’ most signal was used.

The following DNA sequence elements were evaluated: 5) a distal downstream G-rich region, a proximal downstream region enriched in 6) T, 7) GT/TG, and 8) GTGT/TGTG, an intermediate 9) T-rich region, an upstream region enriched in 10) T and 11) TGTA/TATA, and a surrounding 12) AT-rich region. A similar sliding window strategy was utilized, but here the number of windows matching the element to the number of windows not matching the element, i.e. its frequency, was compared to an enrichment threshold value to determine if the given element was considered enriched, encoded 1, or not, encoded (-1). Enrichment thresholds varied across elements. Supplementary Table 9 summarizes the DNA sequence features.

RNA sequencing features.

RNA-Seq features were engineered by defining an upstream region (R1), middle region (R2), and downstream region (R3) for each of the following: 1) intra- and 2) inter-bin. For intra-bin,

the 100 base bin was divided into 34, 33, and 33 bases 5' to 3'. For inter-bin, the 100 bases 5' the 100 base bin, the bin itself, and the 100 bases 3' the bin served as R1, R2, and R3, respectively. The median coverage values of the regions were combined in seven ways for each the intra- and inter-bin to give 14 features:

1) R1-R2
2) R2-R3
3) R1/(R1+R2+R3)
4) R2/(R1+R2+R3)
5) R3/(R1+R2+R3)
6) R2/(R1+R3)
7) R3/(R1+R3)

Note that if the denominator equaled zero, the feature was given a zero.

Supplementary References

1 Arefeen, A., Liu, J., Xiao, X. & Jiang, T. TAPAS: tool for alternative polyadenylation site analysis. *Bioinformatics* **34**, 2521-2529, doi:10.1093/bioinformatics/bty110 (2018).

2 Bogard, N., Linder, J., Rosenberg, A. B. & Seelig, G. A Deep Neural Network for Predicting and Engineering Alternative Polyadenylation. *Cell* **178**, 91-106 e123, doi:10.1016/j.cell.2019.04.046 (2019).

3 Herrmann, C. J. *et al.* PolyASite 2.0: a consolidated atlas of polyadenylation sites from 3' end sequencing. *Nucleic Acids Res* **48**, D174-D179, doi:10.1093/nar/gkz918 (2020).

4 Wang, R., Nambiar, R., Zheng, D. & Tian, B. PolyA_DB 3 catalogs cleavage and polyadenylation sites identified by deep sequencing in multiple genomes. *Nucleic Acids Res* **46**, D315-D319, doi:10.1093/nar/gkx1000 (2018).

5 Ye, C., Long, Y., Ji, G., Li, Q. Q. & Wu, X. APAtrap: identification and quantification of alternative polyadenylation sites from RNA-seq data. *Bioinformatics* **34**, 1841-1849, doi:10.1093/bioinformatics/bty029 (2018).

6 Miura, P., Sanfilippo, P., Shenker, S. & Lai, E. C. Alternative polyadenylation in the nervous system: to what lengths will 3' UTR extensions take us? *BioEssays : news and reviews in molecular, cellular and developmental biology* **36**, 766-777, doi:10.1002/bies.201300174 (2014).
