# Supplementary material for: Aptardi predicts polyadenylation sites in sample-specific transcriptomes using high-throughput RNA sequencing and DNA sequence
Source: Nat Commun. 2021 Mar 12;12:1652. doi: 10.1038/s41467-021-21894-x (PMC7955126; doi:10.1038/s41467-021-21894-x)
Supplement: Supplementary file 3 — Description of Additional Supplementary Files [file 41467_2021_21894_MOESM3_ESM.pdf]

Description of Additional Supplementary Files

File Name: Supplementary Data 1
Description: Transcripts that could be associated with an Ensembl gene symbol and were not differentially expressed (p-value <= 0.001) in the original transcriptome but differentially expressed in the aptardi modified transcriptome.
