# Supplementary material for: Aptardi predicts polyadenylation sites in sample-specific transcriptomes using high-throughput RNA sequencing and DNA sequence
Source: Nat Commun. 2021 Mar 12;12:1652. doi: 10.1038/s41467-021-21894-x (PMC7955126; doi:10.1038/s41467-021-21894-x)
Supplement: Supplementary file 5 — Reporting Summary [file 41467_2021_21894_MOESM5_ESM.pdf]

nature research

Corresponding author(s): Ryan Lusk

Last updated by author(s): Feb 4, 2021

Reporting Summary

Nature Research wishes to improve the reproducibility of the work that we publish. This form provides structure for consistency and transparency in reporting. For further information on Nature Research policies, see our Editorial Policies and the Editorial Policy Checklist.

Statistics

For all statistical analyses, confirm that the following items are present in the figure legend, table legend, main text, or Methods section.

n/a	Confirmed	
☐	☒	The exact sample size (*n*) for each experimental group/condition, given as a discrete number and unit of measurement
☐	☒	A statement on whether measurements were taken from distinct samples or whether the same sample was measured repeatedly
☐	☒	The statistical test(s) used AND whether they are one- or two-sided<br>*Only common tests should be described solely by name; describe more complex techniques in the Methods section.*
☐	☒	A description of all covariates tested
☐	☒	A description of any assumptions or corrections, such as tests of normality and adjustment for multiple comparisons
☐	☒	A full description of the statistical parameters including central tendency (e.g. means) or other basic estimates (e.g. regression coefficient) AND variation (e.g. standard deviation) or associated estimates of uncertainty (e.g. confidence intervals)
☐	☒	For null hypothesis testing, the test statistic (e.g. *F*, *t*, *r*) with confidence intervals, effect sizes, degrees of freedom and *P* value noted<br>*Give P values as exact values whenever suitable.*
☒	☐	For Bayesian analysis, information on the choice of priors and Markov chain Monte Carlo settings
☒	☐	For hierarchical and complex designs, identification of the appropriate level for tests and full reporting of outcomes
☐	☒	Estimates of effect sizes (e.g. Cohen's *d*, Pearson's *r*), indicating how they were calculated

Our web collection on statistics for biologists contains articles on many of the points above.

Software and code

Policy information about availability of computer code

Data collection	The SRA Toolkit (v.2.8.2) was used to download datasets accessed via the NCBI Sequence Read Archive
Data analysis	FastQC (v.0.11.4): Assess quality of RNA sequencing reads<br>cutadapt (v.1.9.1) and trimmomatic (v.0.39): Trim adapters for RNA sequencing reads<br>HISAT2 (v.2.1.0): Align RNA sequencing reads to the genome<br>SAMtools (v.1.9): Convert mapped RNA sequencing reads to a sorted Binary Alignment Map (BAM) file, convert BAM file to bedgraph file (used by aptardi), merge individual BAM files (for rat differential expression analysis)<br>StringTie (v.1.3.5): Generate an original transcriptome<br>RSEM (v.1.2.31): Quantitate transcripts for rat differential expression analysis<br>R (v.4.0.2) and the DESeq2 (v.1.28.1) package: Perform rat differential expression<br>BEDtools (v.2.29.2): Subtract overlapping 3' modified terminal exons (used by aptardi), access DNA sequence (used by aptardi and used to get sequence for APARENT)<br>Keras (v.2.3.1) and TensorFlow (v.2.0.0): Train and deploy machine learning model (used by aptardi)<br>Python3 (v.3.7.7): Programming language for aptardi, perform all other analysis and all visualizations<br>aptardi (v.1.0.0): Software generated (https://github.com/luskry/aptardi)

For manuscripts utilizing custom algorithms or software that are central to the research but not yet described in published literature, software must be made available to editors and reviewers. We strongly encourage code deposition in a community repository (e.g. GitHub). See the Nature Research guidelines for submitting code & software for further information.

Data

Policy information about availability of data

All manuscripts must include a data availability statement. This statement should provide the following information, where applicable:

- Accession codes, unique identifiers, or web links for publicly available datasets
- A list of figures that have associated raw data
- A description of any restrictions on data availability

All data are publicly available. The genomic sequence data that support the findings of this study are available on the UCSC Genome Browser (human: hg38/GRCh38; rat: rn6/Rnor_6.0; mouse: mm10/GRCm38) and PhenoGen (BNLx: BN-Lx/CubPrin; SHR: SHR/OlaIpcvPrin). The polyadenylation sites from PolyA-Seq data that support the findings of this study are available on the UCSC Table Browser. The polyadenylation sites from PolyA_DB and PolyASite 2.0 that support the findings of this study are available on their respective websites. The RNA sequencing data that support the finding of this study are available on the NCBI Sequence Read Archive (Human Brain Reference RNA sequencing: Accession: PRJNA510978, SRA runs: SRR5236425-30; 2nd Human Brain Reference and Universal Human Reference RNA sequencing: Accession: PRJNA362835, 2nd HBR SRA runs: SRR5236425-30, UHR SRA runs: SRR5236455-60; Control vs CFIm25 knockdown RNA sequencing: Accession: PRJNA182153, control SRA run: SRR1238549, CFIm25 knockdown SRA run: SRR1238551; Mouse tissue analysis RNA sequencing: Accession: PRJNA375882, brain SRA runs: SRR5273637 and SRR5273673, liver SRA runs: SRR5273636 and SRR5273672). The BNLx and SHR RNA sequencing that support this study have been deposited in NCBI Sequence Read Archive with the primary accession code GSE166117 (BNLx: GSM5061950-52; SHR: GSM5061947-49).

Field-specific reporting

Please select the one below that is the best fit for your research. If you are not sure, read the appropriate sections before making your selection.

☒ Life sciences ☐ Behavioural & social sciences ☐ Ecological, evolutionary & environmental sciences

For a reference copy of the document with all sections, see nature.com/documents/nr-reporting-summary-flat.pdf

Life sciences study design

All studies must disclose on these points even when the disclosure is negative.

Sample size	To evaluate the applicability of aptardi, we used five datasets. We chose five datasets because this number was sufficient for evaluation, and we chose these datasets in particular because they had sufficient similarities and differences (e.g. tissue, organism, RNA sequencing library preparation) to assess the applicability of the aptardi prediction model. For machine learning, transcripts were split into 60/20/20 training, validation, and testing sets, respectively as is standard in the field.
Data exclusions	Transcript extensions overlapping neighboring transcripts (on the same strand) were truncated to remove overlap. RNA sequencing coverage was used to further truncate transcript extensions similar to other methods employed in the literature. Overall, this was done to prevent arbitrarily extending transcripts when no read coverage exists (i.e. not expressed).
Replication	Replication was assessed in by: 1) evaluating the performance of the aptardi prediction model on the testing set when using five random train-validate-test splits 2) applying the aptardi prediction model across four diverse datasets and 3) building individual prediction models from each of these four alternative datasets. All attempts at replication were successful.
Randomization	Randomization was ensured by using random training, validation, and testing splits when training machine learning models.
Blinding	Blinding is not required as there are no treatment and control groups in our study.

Reporting for specific materials, systems and methods

We require information from authors about some types of materials, experimental systems and methods used in many studies. Here, indicate whether each material, system or method listed is relevant to your study. If you are not sure if a list item applies to your research, read the appropriate section before selecting a response.

Materials & experimental systems

n/a	Involved in the study	
☒	☐	Antibodies
☒	☐	Eukaryotic cell lines
☒	☐	Palaeontology and archaeology
☒	☐	Animals and other organisms
☒	☐	Human research participants
☒	☐	Clinical data
☒	☐	Dual use research of concern

Methods

n/a	Involved in the study	
☒	☐	ChIP-seq
☒	☐	Flow cytometry
☒	☐	MRI-based neuroimaging
